# *Silybum marianum*-Derived Compounds in Prostate Cancer: Mechanisms of Action and Translational Potential

**DOI:** 10.3390/ijms27104605

**Published:** 2026-05-20

**Authors:** Federica Randisi, Giulia Modoni, Mattia Riva, Gianpaolo Perletti, Davide Odorico, Emanuela Marras, Marzia Bruna Gariboldi

**Affiliations:** Department of Biotechnology and Life Sciences (DBSV), University of Insubria, 21100 Varese, Italy; frandisi1@uninsubria.it (F.R.); giulia.modoni@istitutotumori.mi.it (G.M.); mattia.riva@istitutotumori.mi.it (M.R.); gianpaolo.perletti@uninsubria.it (G.P.); dodorico@uninsubria.it (D.O.); emanuela.marras@uninsubria.it (E.M.)

**Keywords:** prostate cancer, *Silybum marianum*, milk thistle, silymarin, silibinin, in vitro studies, in vivo studies, human studies

## Abstract

Prostate cancer (PCa) is the second most frequently diagnosed solid malignancy in men and a major cause of cancer-related mortality worldwide. While localized disease is associated with excellent long-term survival, advanced and castration-resistant PCa continues to represent a major therapeutic challenge. Current management ranges from active surveillance for indolent tumors to multimodal systemic approaches for metastatic disease. In this context, natural compounds are attracting increasing interest as adjunctive or novel therapeutic agents. Among these, silymarin, a *Silybum marianum*-derived flavonolignan complex, has shown promising antineoplastic activity in preclinical PCa models. In vitro, silymarin compounds consistently inhibit PCa cell proliferation by inducing G1 and G2/M cell cycle arrest, upregulating cyclin-dependent kinase inhibitors, and activating caspase-dependent apoptotic pathways. They also modulate key oncogenic signaling pathways involved in cell survival, proliferation, invasion, and metastasis. In vivo xenograft and transgenic models further show reduced tumor growth, angiogenesis, and metastatic spread with limited systemic toxicity. Emerging clinical evidence, including systematic reviews and meta-analyses, suggests translational potential; however, robust randomized trials are needed to define optimal formulations, dosing strategies, and therapeutic efficacy in PCa patients. This review provides a comprehensive overview of the molecular mechanisms, preclinical efficacy, and emerging clinical evidence supporting silymarin as a candidate for future PCa research.

## 1. Introduction

Prostate cancer (PCa) is the second most frequently diagnosed solid-organ malignancy in men and represents a leading cause of cancer-related mortality, accounting for over 350,000 deaths annually worldwide [1,2,3,4]. Its incidence is strongly associated with the Human Development Index (HDI), representing approximately 7% of newly diagnosed cancers globally but rising to 30–35% in countries with high or very high HDI [3,5]. Notably, mortality does not parallel incidence trends: low-HDI countries experience the highest mortality rates, whereas high-income nations have observed a steady decline in PCa-related mortality since the mid-1990s, reflecting advances in early detection, risk stratification, and therapeutic strategies [3,6,7].

At diagnosis, 75–80% of patients present with organ-confined disease, which is associated with a nearly 100% 5-year survival rate [1,2]. Approximately 15% exhibit locoregional metastases and 5% present with distant metastatic disease, often involving bone and lymph nodes. Advanced-stage disease is associated with substantially worse outcomes, with 5-year overall survival declining to around 30% [2].

PCa arises predominantly in the peripheral zone of the prostate [8]. Tumor initiation involves basal or luminal epithelial cells that accumulate genetic and epigenetic alterations, driving malignant transformation [2]. The earliest recognizable precursor lesion, high-grade prostatic intraepithelial neoplasia (HG-PIN), is characterized by aberrant intraglandular epithelial proliferation and significant cellular alterations and is widely considered a putative precursor to invasive adenocarcinoma. Although HG-PIN confers an increased risk for adenocarcinoma development and can sometimes be present in the gland together with frank carcinoma, progression to clinically manifest cancer may occur over a prolonged latency period, sometimes spanning a decade [9].

At the molecular level, early-stage PCa is characterized by recurrent genomic alterations. The most prevalent include gene fusions between transmembrane protease serine 2 (TMPRSS2) and members of the erythroblast transformation-specific (ETS) transcription factor family, particularly ERG. The TMPRSS2–ERG fusion is detected in up to 60% of primary tumors and represents a hallmark molecular event in PCa pathogenesis. Additional recurrent alterations include loss-of-function mutations in SPOP and activating mutations in FOXA1, underscoring the molecular heterogeneity of early disease [2,8].

Disease progression to metastatic prostate cancer is marked by increased mutational burden and a higher prevalence of copy number alterations. Metastatic disease initially manifests as metastatic castration-sensitive prostate cancer (mCSPC). However, under the selective pressure of androgen-deprivation therapy (ADT), which represents the standard treatment for advanced disease, tumors commonly develop therapeutic resistance and transition to metastatic castration-resistant prostate cancer (mCRPC) [10,11]. This stage is characterized by alterations affecting the androgen receptor (AR) signaling axis, including AR amplification and gain-of-function mutations, as well as dysregulation of transcriptional regulators such as FOXA1. Inactivation of tumor suppressor genes, including TP53, RB1, and PTEN, is common, as are defects in homologous recombination repair genes such as BRCA2 and ATM, contributing to genomic instability and therapeutic vulnerabilities [11,12].

Only three non-modifiable risk factors for PCa are consistently supported by strong epidemiological evidence: advancing age, ethnicity, and family history. Incidence increases sharply after age 55, peaking between 70 and 74. Significant ethnic disparities remain, with African American men experiencing about a 60% higher incidence and increased disease-specific mortality compared to Caucasian men. Additionally, a positive family history further raises the risk, with first-degree relatives associated with roughly double the likelihood. BRCA2 pathogenic variants represent an even more substantial inherited predisposition, as highlighted by a meta-analysis by Nyberg et al., which identified BRCA2 as a strong genetic risk factor for prostate cancer [13].

Lifestyle and environmental factors have been extensively studied, though their causal roles are less clearly established. The high incidence of PCa in Western countries has linked Western dietary patterns to prostate cancer development. Epidemiological and preclinical data indicate that high intake of saturated fats, dairy products and charred red meat may promote tumor growth, partly by producing carcinogenic compounds such as polycyclic aromatic hydrocarbons and heterocyclic aromatic amines (e.g., 2-amino-1-methyl-6-phenylimidazo[4,5-b]pyridine), which can increase genetic instability. While obesity and tobacco use are known risk factors for several cancers, their connection to PCa remains inconsistent. Nonetheless, both are independently associated with higher PCa-specific mortality and worse clinical outcomes. Conversely, regular physical activity and diets rich in bioactive compounds, including lycopene, selenium, and cruciferous vegetables, have been linked to a modest decrease in overall risk and a lower chance of lethal disease [14,15].

Clinical management of PCa requires a comprehensive, risk-adapted, and patient-centered approach integrating clinical, pathological, and molecular parameters. Key determinants include plasma levels of markers such as the prostate-specific antigen (PSA), TNM stage, Gleason/ISUP grade, and castration-sensitive versus castration-resistant status. In addition, molecular tumor features and patient-related factors, such as life expectancy, comorbidities, and patient preferences, further inform therapeutic decision-making, forming the basis of contemporary personalized care [16,17].

Low-risk localized PCa is generally managed with active surveillance, as treatment-related morbidity may outweigh clinical benefit, with definitive local therapy, such as radical prostatectomy or radiotherapy, with or without androgen-deprivation therapy (ADT), reserved for disease progression [18]. In contrast, metastatic PCa relies on systemic ADT, historically combined with docetaxel plus prednisone in advanced stages [19]. In recent years, however, the therapeutic landscape has expanded considerably with the introduction of next-generation androgen receptor signaling inhibitors (ARSIs), poly (ADP-ribose) polymerase inhibitors (PARPi), novel taxanes, and bone-targeted radionuclides [20]. However, real-world implementation remains suboptimal, with fewer than half of patients with metastatic castration-resistant PCa (mCRPC) receiving guideline-concordant therapy, largely due to uncertainties in treatment sequencing and limited comparative evidence [21]. Treatment-related toxicities, particularly cardiovascular complications, further complicate management [22]. Optimizing outcomes, therefore, depends on precise risk stratification, early identification of aggressive disease phenotypes, and broader adoption of molecular profiling [23]. Despite clear guideline recommendations, genomic testing in mCRPC remains underutilized, limiting the full realization of personalized therapeutic strategies [24].

Extensive evidence indicates that natural products can selectively modulate multiple molecular targets and signaling pathways involved in tumor development and progression [25,26]. Among these, polyphenols have been demonstrated to interfere with AR stability and transcriptional activity, thereby attenuating downstream pro-survival signaling pathways critical for aggressive PCa growth [27,28]. Phytochemicals have also been shown to suppress metastatic potential by reversing epithelial-to-mesenchymal transition (EMT) and downregulating matrix metalloproteinases, thus reducing tumor cell invasion [29], and inducing programmed cell death across various PCa cellular models, including LNCaP, PC-82, and DU145 cell lines [30,31]. Furthermore, they trigger non-canonical, caspase-independent pathways such as paraptosis in DU145 and PC3 cells, offering a potential strategy to bypass conventional apoptotic resistance [32].

Recently, bioactive constituents of *Silybum marianum* (milk thistle), including flavonoids, flavolignans, and dihydroflavonol, have been explored for their biological activities, including antioxidant, anti-inflammatory, organ-protective, and immunomodulatory effects [33,34,35]. In addition, their anticancer potential was evaluated in several experimental models, including PCa cell lines [36]. Silymarin, the standardized extract of *Silybum marianum*, with silibinin as its most active constituent, has demonstrated significant activity in vitro and in vivo. Mechanistically, silybin induces cytoskeletal disorganization by modulating the focal adhesion kinase (FAK)/Src signaling pathway, thereby impairing cellular adhesion and motility [37]. Furthermore, silibinin reprograms tumor cell metabolism by activating AMP-activated protein kinase (AMPK), thus suppressing de novo lipogenesis and reducing intracellular lipid and cholesterol accumulation, processes essential for membrane biosynthesis and tumor energy homeostasis [38,39].

This review presents a comprehensive overview of the in vitro and in vivo effects of *Silybum marianum*, with particular emphasis on its potential therapeutic applications in PCa. Consideration is also given to the evaluation of clinical studies and meta-analyses investigating the use of silymarin derivatives in human subjects.

## 2. *Silybum marianum* Constituents and Activities

Silymarin, the principal bioactive complex extracted from *Silybum marianum* (milk thistle), is primarily derived from the plant’s seeds, although it is also detectable in the leaves and fruits [40]. It comprises approximately 70–80% flavonolignans, structurally characterized as hybrid molecules formed through oxidative coupling between the flavanonol taxifolin and the phenylpropanoid coniferyl alcohol. Their core architecture consists of a typical flavanonol scaffold fused to a lignan-derived unit via a dioxane or benzodioxane ring system (Table 1).

The predominant flavolignans of silymarin are silybin (also referred to as silibinin), isosilybin, silychristin, isosilychristin, and silydianin, together with minor amounts of flavonoids, including taxifolin, quercetin, and apigenin. The remaining 20–30% consist of a heterogeneous, partially characterized polymeric flavonoid fraction [35](16). Except for silydianin, flavonolignans in silymarin occur as diastereomeric pairs, conventionally designated as A and B isoforms, present in variable proportions. Among these constituents, silybin represents the most abundant and pharmacologically active component of silymarin and is widely regarded as the major contributor to its therapeutic properties, owing to its superior bioactivity compared with other flavonolignans [36](17).

Besides being a constituent of *Silybum marianum*, silibinin has also been reported in *Silybum eburneum*. Interestingly, certain fungal endophytes growing on the surface of plants of the *Silybum* genus, such as the fungus *Aspergillus iizukae*, were found to contain flavolignans of the host plant, such as silybin and isosilybin [41].

**Figure 1:**
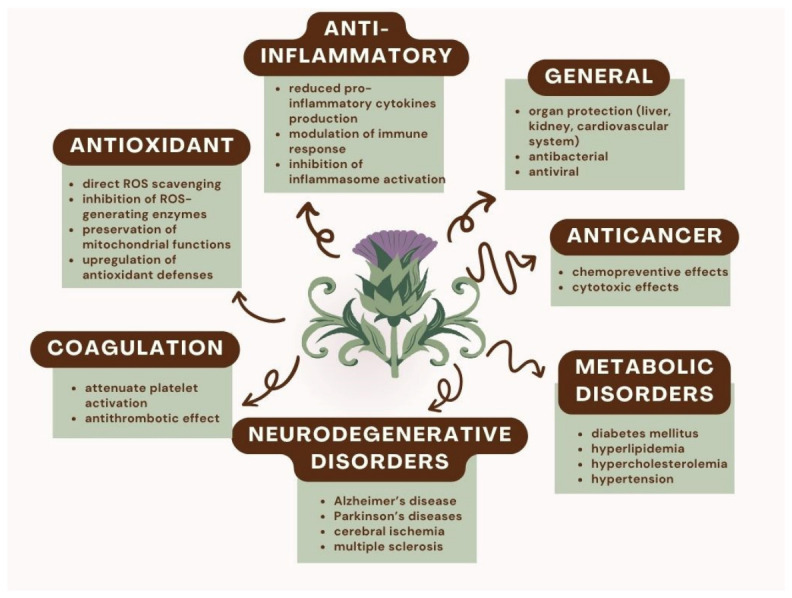
Silymarin/silibinin activities.

Silymarin and its purified derivatives exhibit a wide range of biological activities supporting their extensive therapeutic potential (Figure 1). Their pharmacological effects include antioxidant, anti-inflammatory, anticancer, and chemopreventive properties, as well as cytoprotective actions [42]. At the molecular level, the antioxidant activity of *Silybum marianum*’s components is mediated through multiple complementary mechanisms, including direct scavenging of reactive oxygen species (ROS), inhibition of ROS-generating enzymes, preservation of mitochondrial function, and upregulation of endogenous antioxidant defenses via activation of redox-sensitive transcription factors and phase II detoxifying enzymes [43,44,45]. Through these coordinated actions, silymarin and its derivatives contribute to maintaining cellular redox homeostasis. Beyond their antioxidant capacity, they exert anti-inflammatory activity by modulating several key inflammation-related signaling pathways. Their activity is associated with reduced production of pro-inflammatory cytokines through regulation of cytokine-mediated signaling cascades, modulation of immune cell responses, suppression of endotoxin-induced cytokine release, and inhibition of inflammasome activation [46]. Given the pivotal role of oxidative stress and chronic inflammation in the pathogenesis of numerous disorders, the ability of *Silybum marianum* components to attenuate these interconnected processes supports their potential utility in the prevention and management of a broad range of conditions, including liver diseases, cardiovascular disorders, and cancer [44,47].

Silymarin has been extensively investigated for its organ-protective effects, particularly in the liver, renal, cardiovascular, and neural tissues, as well as for its antiviral and antibacterial activities [48,49]. Notably, silymarin is among the most widely utilized bioactive constituents in dietary supplements for the management of hepatotoxicity and chronic liver disorders [34]. Its hepatoprotective activity is primarily attributed to the inhibition of toxin uptake into hepatocytes, thereby preventing toxin-induced cellular injury, necrosis, and ferroptosis, as well as to the suppression of lipid peroxidation and oxidative stress-mediated hepatic damage [50]. In addition, silymarin promotes liver regeneration by stimulating hepatocyte proliferation and has demonstrated antifibrotic effects by attenuating hepatic stellate cell activation and extracellular matrix deposition [51].

Beyond liver disease, accumulating evidence supports the therapeutic relevance of silymarin in metabolic disorders, including diabetes mellitus and its associated complications, as well as hyperlipidemia and hypercholesterolemia [52,53,54]. Its metabolic benefits encompass antihyperglycemic, hypolipidemic, anti-atherosclerotic, and antihypertensive effects. Mechanistically, silymarin contributes to improved glycemic control by reducing fasting blood glucose levels, enhancing insulin sensitivity, and mitigating insulin resistance. Concurrently, it favorably modulates lipid profiles by decreasing low-density lipoprotein (LDL) cholesterol and increasing high-density lipoprotein (HDL) cholesterol while also exerting protective effects against endothelial dysfunction [55,56]. Moreover, silymarin has been shown to influence immune responses, regulate hormone functions, and interfere with multidrug resistance mechanisms, demonstrating its ability to target multiple connected cellular pathways [57,58].

Owing to these pleiotropic biological properties, silymarin has been investigated as a potential therapeutic agent across a wide range of pathological conditions. Preclinical and clinical studies have explored its role in neurodegenerative disorders, including Alzheimer’s disease, Parkinson’s disease, cerebral ischemia, and multiple sclerosis, as well as in viral infections such as SARS-CoV-2 [59,60,61,62,63,64,65,66,67,68]. In particular, the neuroprotective potential of silymarin has been attributed to its ability to attenuate oxidative stress within the central nervous system and to modulate molecular pathways implicated in β-amyloid aggregation, neuroinflammation, and estrogen receptor-mediated neuronal apoptosis. In experimental models, silymarin has also demonstrated beneficial effects on psychomotor performance and cognitive function, reinforcing its potential relevance in neurodegenerative conditions [60,69].

In addition, accumulating data indicate that silybin can inhibit serine proteases involved in the coagulation cascade and attenuate platelet activation, thereby exerting antiplatelet and antithrombotic effects. These findings have generated increasing interest in silybin as a candidate for developing novel antithrombotic strategies [70,71,72].

## 3. Anticancer Effects of *Silybum marianum* Constituents

Accumulating preclinical evidence suggests that silymarin, and particularly its major bioactive constituent silibinin, exerts significant anticancer activity across a broad spectrum of malignancies, including colorectal, bladder, breast, gastric, prostate, and lung cancers; hepatocellular carcinoma; glioblastoma; laryngeal and skin cancers; and hematological malignancies such as leukemia [58,73]. Its antineoplastic effects are mediated through multiple complementary mechanisms, including induction of apoptosis, cell cycle arrest, upregulation of cyclin-dependent kinase inhibitors (CDKIs), and modulation of inflammatory transcription factors. In addition, silymarin regulates the expression of genes involved in tumor cell proliferation, invasion, metastasis, and angiogenesis [74,75].

A central mechanism underlying the antiproliferative activity of silibinin involves modulation of the mitogen-activated protein kinase (MAPK) signaling cascade [58,76]. The canonical RAS–RAF–MEK–ERK axis governs essential cellular processes, including proliferation, differentiation, and survival, and its dysregulation is a hallmark of tumorigenesis [77]. Silibinin suppresses MAPK/ERK1/2 signaling while enhancing stress-activated pathways such as c-Jun N-terminal kinase (JNK) and p38 MAPK, thereby shifting the balance from pro-survival to pro-apoptotic signaling [78]. Consistently, silibinin downregulates anti-apoptotic proteins (Bcl-2, Bcl-xL, and survivin) and upregulates pro-apoptotic mediators such as Bax, activating caspase-9 and caspase-3 [79,80]. Furthermore, it stimulates death receptor-mediated apoptosis by upregulating DR4/DR5 and TRAIL expression, supporting the concurrent activation of intrinsic and extrinsic apoptotic pathways [81,82].

Silibinin also exerts marked effects on cell cycle regulation. Cyclin-dependent kinases (CDKs) and their cyclin partners are key drivers of cell cycle progression and are frequently dysregulated in cancer [83]. Silymarin and its flavonolignans inhibit CDK overexpression in a dose-dependent manner, promoting antitumor activity [84]. Lower concentrations of silibinin induce G1-phase arrest, whereas higher doses result in G2/M arrest. These effects are associated with enhanced interaction between CDKIs and CDKs and reduced CDK activity, thereby suppressing uncontrolled proliferation [85,86].

Beyond MAPK and cell cycle control, silibinin modulates additional oncogenic pathways involved in survival and regulated cell death. Notably, it inhibits the phosphatidylinositol-3-kinase/protein kinase B/mammalian target of rapamycin (PI3K/Akt/mTOR) axis, a central signaling network that integrates metabolic and growth-related cues and is frequently hyperactivated in cancer [87]. Both in vitro and in vivo studies demonstrate that silymarin suppresses tumor growth by inhibiting PI3K/Akt/mTOR signaling [88]. Moreover, silibinin impedes PI3K activity and downregulates Forkhead box M1 (FoxM1), thereby activating the mitochondrial apoptotic pathway [89]. Silymarin further interferes with the Janus kinase/signal transducer and activator of transcription (JAK/STAT) pathway, a critical regulator of inflammation, immune responses, and tumor progression [90]. By reducing phosphorylation of JAK2 and STAT3 and preventing STAT3 nuclear translocation and DNA binding, silibinin suppresses STAT3-dependent gene transcription and promotes tumor cell death [91].

Additionally, silibinin targets epidermal growth factor receptor (EGFR) and nuclear factor κB (NF-κB) signaling, which frequently cooperate to sustain oncogenic processes in solid tumors. EGFR activation promotes NF-κB-mediated transcription of genes involved in proliferation, survival, and inflammation [92]. Silibinin inhibits EGFR phosphorylation and downstream signaling, thereby attenuating EGFR-driven oncogenic pathways and its crosstalk with the NF-κB signaling pathway [93].

Silibinin inhibits histone deacetylase (HDAC) activity and downregulates HDAC1–3, thereby increasing global acetylation of histones H3 and H4. In combination with HDAC inhibitors (HDACi), silibinin synergistically enhances cytotoxicity, an effect associated with increased p21 expression [94]. Since aberrant HDAC activity promotes chromatin condensation and transcriptional silencing of tumor suppressor genes, its modulation represents an important mechanism through which silibinin may counteract cancer cell proliferation and survival [95].

Several in vitro studies have shown that silibinin inhibits cancer cell migration, invasion, and adhesion in a dose- and time-dependent manner. These effects are associated with reversal of the epithelial–mesenchymal transition (EMT) phenotype through the downregulation of vimentin and matrix metalloproteinase-2 (MMP-2). In addition, silibinin inhibits nuclear factor kappa-B (NF-κB) nuclear translocation, leading to reduced expression of EMT-related transcription factors such as ZEB1 and SLUG [96,97]. Consistent with these findings, in vivo studies in mouse models have shown that silibinin decreases the expression of vascular endothelial growth factor (VEGF), VEGF receptor-2 (VEGFR-2), and basic fibroblast growth factor (bFGF), while increasing E-cadherin levels and reducing vimentin and the EMT regulator Snail-1 in tumor tissues [58].

The antitumor activity of silibinin is also partly mediated by immune system modulation. In vivo studies have demonstrated that silibinin reduces the accumulation of myeloid-derived suppressor cells in the tumor microenvironment while promoting T-cell infiltration [98]. Moreover, through its antioxidant properties, silibinin attenuates ROS production and supports leukocyte function. It also inhibits NF-κB signaling, thereby reducing the expression of pro-tumorigenic cytokines such as interleukin-6 (IL-6), tumor necrosis factor-α (TNF-α), and transforming growth factor-β1 (TGF-β1), which suppress cytotoxic T lymphocyte (CTL) activity [99]. In addition, silibinin has been reported to induce immunogenic cell death (ICD), enhancing antitumor immune responses and the immunogenicity of chemotherapy [100].

Silibinin has been shown to enhance chemotherapy-induced cytotoxicity and sensitize chemoresistant cancer cells to anticancer drugs [101]. This effect is partly mediated by the inhibition of P-glycoprotein-dependent drug efflux, thereby overcoming multidrug resistance [102]. Furthermore, silibinin interferes with TGF-β signaling by preventing SMAD2/3 phosphorylation and repressing SMAD-dependent transcription, counteracting EMT-associated resistance to tyrosine kinase inhibitors [103].

In addition, silybin exerts chemoprotective effects by modulating xenobiotic-metabolizing enzymes. Specifically, it inhibits phase I enzymes while inducing phase II detoxifying enzymes, thereby reducing the formation of reactive intermediates and promoting their detoxification [104].

Finally, silymarin and its bioactive constituents have also been investigated for the prevention of several adverse effects associated with anticancer therapies, including capecitabine-induced hand–foot syndrome, cisplatin-induced nephrotoxicity, and radiation-induced mucositis and dermatitis [105,106,107].

**Figure 2:**
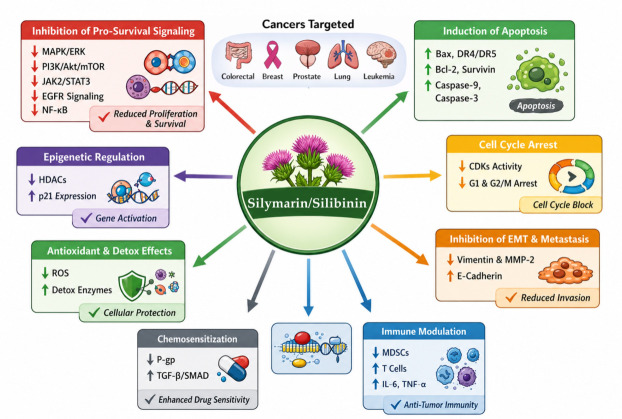
Summary of the effects of silymarin and silibinin on cancer cells and the molecular mechanisms and pathways involved.

A summary of the anticancer effects of silymarin and silibinin, along with the involved molecular mechanisms and pathways, is reported in Figure 2.

## 4. Effects of *Silybum marianum* on Prostate Cancer

As previously mentioned, silymarin and its pharmacologically active constituents have gained considerable attention for their potential to inhibit tumor progression, particularly in prostate cancer [36,108]. Among these compounds, silibinin exerts significant antitumor effects in prostate cancer by modulating key molecular pathways regulating cell proliferation, apoptosis, invasion, metastasis, and angiogenesis [108]. Over the past few decades, several in vitro and in vivo studies have investigated the anticancer efficacy of silibinin in experimental models of prostate cancer. Table 2 summarizes the main studies in this area, highlighting the bioactive compounds investigated and the molecular mechanisms proposed to explain their antineoplastic activity.

### 4.1. In Vitro Studies

Most in vitro studies investigating the anticancer activity of milk thistle-derived compounds in prostate cancer models have focused primarily on silibinin and its parent extract, silymarin. In agreement with the general antiproliferative mechanisms already described in other tumor models, both agents suppress the proliferation of PC-3 prostate cancer cells by inducing G1 and G2/M cell cycle arrest and activating caspase-dependent apoptotic pathways [86]. In prostate cancer cells, G1 phase arrest is characterized by the downregulation of G1-associated cyclins and cyclin-dependent kinases, together with the induction of the CDK inhibitors Cip1/p21 and Kip1/p27, thereby impairing G1-to-S phase transition. In parallel, G2/M arrest involves the modulation of the Chk2–Cdc25C–Cdc2/cyclin B1 signaling axis [109,110]. These effects are accompanied by reduced PSA expression, further supporting the inhibitory activity of silibinin on prostate cancer cell growth and progression [110].

Silibinin has also been reported to induce autophagy in prostate cancer PC-3 cells, as demonstrated by increased LC3-II expression, the accumulation of acidic vesicular organelles (AVOs), and the formation of GFP-LC3 aggregates. Mechanistically, ROS appear to mediate autophagy induction in this model. Notably, the pharmacological inhibition of autophagy potentiates silibinin-induced apoptosis, suggesting that autophagy may act as a cytoprotective adaptive response that modulates the balance between cell survival and cell death in prostate cancer cells [111]. Consistent with observations previously reported in other tumor types, silibinin also interferes with signaling pathways involved in tumor progression, migration, and invasion. Under hypoxic conditions, silibinin attenuates HIF-1α-mediated signaling in LNCaP cells, thereby suppressing lipogenesis, clonogenic growth, and NOX activity, all of which contribute to prostate cancer progression and angiogenesis [38]. In the same experimental setting, silibinin inhibits prostate cancer cell migration and invasion [97]. Similar effects have been described in PC-3 and highly bone-metastatic ARCaP(M) cells and are associated with reduced vimentin expression, the inhibition of EMT-related pathways, and decreased matrix metalloproteinase levels [96,97,112]. The upregulation of E-cadherin has also been identified as a key mediator of these effects, reinforcing cell–cell adhesion and limiting metastatic potential [113]. Furthermore, silibinin inhibits Wnt/β-catenin signaling in PC-3 and DU145 cells through the repression of the expression and phosphorylation of the Wnt co-receptor LRP6, identifying silibinin as a small-molecule inhibitor of Wnt/LRP6 signaling, a pathway critically involved in prostate cancer progression [114].

### 4.2. In Vivo Studies (Animal Models)

Several studies have shown that silymarin, and, in particular, silibinin, effectively suppresses prostate cancer growth in animal models, consistent with the manifold mechanisms observed in in vitro studies. A preclinical investigation, employing the Transgenic Adenocarcinoma of the Mouse Prostate (TRAMP) model, demonstrated that long-term dietary exposure to silibinin significantly limits disease progression at multiple stages. This effect was reflected by the reduced histopathological severity and malignant potential of prostatic lesions, achieved through the inhibition of tumor cell proliferation, the suppression of angiogenesis, and the attenuation of metastatic dissemination. At the molecular level, silibinin treatment was associated with the downregulation of angiogenesis- and hypoxia-related mediators, including PECAM-1/CD31, VEGF and its receptor VEGF-R2, HIF-1α, and iNOS, as well as a decreased expression of invasion- and EMT-associated molecules such as matrix metalloproteinases, Snail-1, and fibronectin [115,116].

In agreement with these findings, silibinin also exhibited marked chemopreventive and therapeutic effects in human prostate cancer xenograft models. In PC-3 tumor-bearing mice, dietary silibinin significantly inhibited tumor growth without inducing systemic toxicity. Tumor suppression was accompanied by the coordinated modulation of key molecular regulators governing proliferation, survival, and angiogenesis, including the upregulation of IGFBP-3, Cip1/p21, Kip1/p27, and phosphorylated ERK1/2, along with the downregulation of VEGF, Bcl-2, and survivin. Collectively, these molecular alterations favored cell cycle arrest, enhanced apoptosis, and reduced tumor vascularization [117,118].

Further supporting its in vivo antitumor activity, silibinin administered by oral gavage also significantly inhibited tumor growth in orthotopically implanted human PC-3 prostate tumors, indicating that its efficacy extends beyond transgenic models to clinically relevant, human-derived prostate cancer systems. Mechanistically, tumor growth inhibition involved a multi-targeted signaling response, characterized by enhanced ERK1/2 activation, the induction of the CDK inhibitors Cip1/p21 and Kip1/p27, and the concomitant suppression of VEGF, Bcl-2, survivin, and additional oncogenic signaling mediators, including JNK, p38MAPK, Akt, and STAT1/3/5. These findings further corroborate silibinin’s capacity to inhibit proliferation, induce apoptosis, and suppress angiogenesis in vivo [117,119].

Beyond silibinin, other constituents of the silymarin complex have also demonstrated antitumor efficacy in prostate cancer models. Notably, in human DU145 xenografts, isosilibinin significantly inhibited tumor growth, accompanied by the reduced expression of proliferation markers such as PCNA, the suppression of angiogenesis as indicated by decreased CD31 and VEGF levels, and increased apoptosis. Importantly, these antitumor effects persisted even after treatment withdrawal, suggesting a sustained therapeutic benefit. Comparative analyses indicated that isosilibinin may exhibit efficacy comparable to, or slightly greater than, that of silymarin and silibinin, supporting its further investigation as a multi-targeted chemopreventive and therapeutic agent for prostate cancer [118].

A summary of the key findings from in vitro (cell-based) and in vivo (animal-based) studies on the use of silymarin and its components in prostate cancer, along with the potential mechanisms underlying their effects, is presented in Table 2.

**Table 2:** Overview of the major in vitro and in vivo studies investigating the anticancer effects of silymarin and its key components in prostate cancer, including the underlying molecular mechanisms.

Type of Model	Treatment	Potential Mechanisms	Effects	References
In vitro				
PC-3 cells	Silymarin and silibinin (50–100 mg/mL)	Cyclin D1, D3, and E reduction; increase in CDKIs; inhibition of the Chk2–Cdc25C–Cdc2/cyclin B1 pathway	G1 and G2-M cell cycle arrest	[86]
DU145 cells	Silymarin	Induction of CDKIs Cip1/p21 and Kip1/p27; reduction in CDK activity; inhibition of erbB1 (EGFR) activation	Strong inhibition of cell cycle progression	[109]
LNCaP cells	Silibinin	Downregulation of cyclin D1, CDK4, and CDK6; upregulation of Cip1/p21 and Kip1/p27; PSA reduction	Decreased PSA expression, inhibition of cell growth, G1 cell cycle arrest	[110]
PC-3 cells	Silibinin	Upregulation of LC3-II; formation of AVO; GFP-LC3 complexes and ROS	Induction of autophagy	[111]
LNCaP cells	Silibinin	Reduction in HIF-1α expression; NOX activity and lipogenesis	Reduced cell proliferation, inhibited hypoxia-induced lipid accumulation and endothelial tube formation	[38]
PC-3 cells	Silibinin	Reduction in cell adhesion to ECM	Inhibition of cell viability, adhesion and migration	[97]
ARCaP(M), LNCaP, PC-3 and DU145 cells	Silibinin at 50, 100, 200 µM	Reduction in vimentin and MMP-2 expression	Inhibition of cell invasion, motility and migration	[96]
PC-3 and DU145 cells	Silibinin	Repression of LRP6 expression, blockade of LRP6 phosphorylation and inhibition of Wnt/β-catenin signaling	Decreased proliferation	[114]
In vivo				
TRAMP mice	Silibinin-supplemented diet (1%) for 8–15 weeks	Reduction in PECAM-1/CD31, VEGF, VEGFR2, HIF-1α, iNOS, MMPs, Snail-1, and fibronectin	Reduced severity of prostatic lesions, inhibition of angiogenesis and reduction in metastasis to distant organs	[115]
TRAMP mice	Silybin-phytosome (0.5% and 1% *w*/*w* in diet) for 11 weeks	Reduction in microvessel density, VEGF, VEGFR2, plasma VEGF, bFGF, MMPs, Snail-1, and Vimentin; increase in E-cadherin expression	Inhibited tumor growth, prevented progression from PIN to adenocarcinoma, reduced invasion of seminal vesicle, reduction in distant metastasis	[116]
Athymic male mice with orthotopically implanted PC-3 human prostate tumors	Silibinin, 100 mg/kg body weight daily for 7 weeks	Reduction in CDK2, CDK4, CDK6, CDC2, cyclins D1, D3, E, A, VEGF, JNK1/2, and p38MAPK; Akt phosphorylation; inhibition of STAT1/3/5 phosphorylation; upregulation of ERK1/2 phosphorylation and caspase-3 cleavage	Reduction in tumor/urogenital weight, cell proliferation, suppression of tumor vascularization	[119]
Athymic nude mice with human DU145 prostate cancer xenografts	Isosilibinin (50:50 mixture of isosilybin A and B), 200 mg/kg body weight per day for 53 days; compared with silymarin and silibinin	Altered expression of cyclins and CDKs; downregulation of VEGF and PCNA	Significant inhibition of tumor growth, reduction in angiogenesis	[118]

### 4.3. Silymarin and Silybum flavolignans in Prostate Cancer Clinical Studies

Clinical interest in silymarin and its principal bioactive components has grown following early clinical trials exploring their therapeutic potential in human disease. Initial clinical evidence of silymarin compounds has primarily focused on liver-related disorders, such as chronic hepatitis, alcoholic liver disease, and toxin-induced hepatic injury, reflecting well-documented hepatoprotective and antioxidant properties [120,121].

Beyond hepatology, preliminary clinical investigations have also examined metabolic disorders, cancer chemotherapy-related conditions and inflammatory diseases, although the evidence remains heterogeneous [55,122].

Clinical investigations of *Silybum marianum* derivatives have predominantly addressed their role in supportive oncology care, rather than direct antineoplastic activity. In particular, a preparation standardized to 80 mg of silibinin (Silybin A and B) has demonstrated hepatoprotective effects in pediatric patients receiving methotrexate-based chemotherapy for acute lymphoblastic leukemia, with randomized trials reporting improvements in liver function without compromising therapeutic efficacy [123]. Similarly, oral silymarin (140 mg tablets) administered in a triple-blind, placebo-controlled trial of non-metastatic breast cancer patients receiving doxorubicin-based chemotherapy significantly reduced ultrasonographic evidence of fatty liver and improved liver enzyme trends, indicating a preventive effect against chemotherapy-induced hepatotoxicity [124].

This research trajectory has expanded to include emerging clinical trials in prostate cancer (PCa).

A research group at the University of Colorado and the Health Sciences Center conducted a phase I study, followed by a phase II trial, involving prostate cancer patients.

The phase I trial of oral silybin-phytosome included thirteen patients with advanced prostate cancer. The aim was to assess the compound safety, tolerability, and pharmacokinetics and to identify a recommended phase II dose [125]. Patients received escalating high doses of silybin-phytosome (2.5 to 20 g daily) for 4-week courses. The most frequent adverse events were asymptomatic liver effects, particularly grade-2 elevations in bilirubin at the 15 and 20 g dose levels. There was a single instance of grade 3 transaminase elevation (ALT), and no grade 4 toxicities were seen. Minor creatinine elevations were also observed. Exploratory analysis failed to detect objective responses as assessed by prostate-specific antigen (PSA) levels. The authors concluded that 13 g daily in divided doses was a feasible and recommended dose for the phase II study, with liver toxicity being the dose-limiting concern.

The phase II trial was a nonrandomized, prospective, open-label controlled study investigating the pharmacodynamic and tissue bioavailability effects of pre-surgery high-dose oral silybin-phytosome in men with clinically localized adenocarcinoma of the prostate who were candidates for surgical resection of the gland [126]. Subjects in the active arm (*n* = 6) received 13 g/day of silybin-phytosome for 14–31 days prior to prostatectomy. Control patients (*n* = 6) were left untreated. Pharmacokinetic profiling demonstrated transient systemic exposure with mean 1 h post-dose plasma silybin peak concentrations of about 19 µM. However, prostate tissue concentrations were markedly lower and were detected in only three patients (maximum observed concentration: 496.6 pmol/g), indicating minimal prostatic penetration despite elevated systemic levels. Notably, higher prostate concentrations of silybin were assessed in experiments performed in mice (~10 μmol/g) [127]. No significant differences in pre- vs. post-therapy PSA levels were recorded in the silybin group (baseline, 5.4 ng/mL; post-therapy, 5.1 ng/mL), and biomarker analysis revealed no significant modulation of circulating IGF-I or IGFBP-3, suggesting minimal systemic endocrine effects over the short presurgical window. The intervention was well tolerated, with predominantly mild gastrointestinal adverse events and transient grade-2 hyperbilirubinemia in one subject. A single grade 4 postoperative thromboembolic event occurred.

Two double-blind, placebo-controlled trials were performed to test the efficacy of combined formulations, including silymarin, in PCa patients previously subjected to radical prostatectomy.

The 2005 randomized, double-blind, placebo-controlled crossover study by Schröder et al. randomized 49 men with rising PSA after prostatectomy (*n* = 34) or radiotherapy (*n* = 15) to receive a dietary supplement including multiple nutraceuticals (silymarin, soy, isoflavones, and lycopene) or placebo for two 10-week treatment periods separated by a 4-week washout interval [128]. The primary endpoints were the slope of the PSA concentration-time curve (µg/L*d) and PSA doubling time (PSADT). Per-protocol analysis of the results showed that either the slope of the untransformed total serum PSA concentration (*p* = 0.030) or the slope of the log-transformed PSA concentration (*p* = 0.041) was significantly lower during supplement treatment vs. placebo. However, intent-to-treat analysis did not show significant slope differences. PSADT increased ~2.6-fold, from 445 days on placebo to 1150 days on supplement, indicating a slower PSA rise. Thus, a well-tolerated dietary supplement containing silymarin significantly slowed biochemical disease progression (PSA slope and PSADT) in men with rising PSA. However, it is not possible to quantify the potential contribution of silymarin to the therapeutic effect of this multi-component treatment.

The 2010 randomized, double-blind, placebo-controlled trial by Vidlar et al. was aimed at assessing the safety and biochemical effects of daily supplementation with 570 mg silymarin tablets + 240 µg selenium versus placebo in 37 PCa patients following radical prostatectomy [129]. The silymarin-selenium combination improved QoL scores and reduced LDL and total cholesterol compared with the placebo. Undetectable levels of PSA were documented after prostatectomy as well as at the end of treatment in both groups. Thus, no statistically significant intergroup differences in PSA progression were reported. Silymarin was found to be safe and altered metabolic markers associated with prostate cancer biology in combination with selenium. Specific PSA outcomes were not reported as significant, leaving the direct effect of the treatment on PSA progression uncertain.

Nonmalignant prostate conditions are beyond the scope of this review; however, it is worth mentioning that in two double-blind randomized trials conducted in men with benign prostatic hyperplasia, silymarin-based nutraceutical combinations were evaluated for their effects on PSA modulation. In the Valipour et al. 2024 study, 80 men treated for 3 months with silymarin alone or combined with cholecalciferol showed significant improvements in urodynamic parameters, but neither total nor free PSA changed significantly from baseline in any group (*p* = 0.071 and *p* = 0.11, respectively), indicating no measurable short-term PSA modulation [130]. In contrast, the Vostalova et al. 2013 trial, which randomized 55 men to selenium–silymarin or placebo for 6 months, demonstrated a modest yet statistically significant reduction in total PSA in the active-treatment group compared with placebo (*p* < 0.05) [131].

Silybin was included in a recent systematic review and network meta-analysis (NMA) aimed at comparing the therapeutic impacts of diverse natural extract interventions on key biomarkers of prostate cancer progression [132]. Primary outcomes were changes in biomarkers implicated in PCa genesis and progression, including PSA, insulin-like growth factor-1 (IGF-1), and IGF-binding protein-3 (IGFBP-3). Effect sizes were ranked using the surface under the cumulative ranking curve (SUCRA) probabilities. Cluster analysis evaluated comparative efficacy across the three endpoints.

The combination of silybin with selenium emerged as an effective intervention for reducing serum PSA levels among the evaluated natural extracts, with a high SUCRA ranking (~74%). In addition, silybin monotherapy demonstrated the highest probability (~84.6%) of lowering serum IGF-1, a growth factor associated with PCa proliferation. Silybin alone also ranked highest (~67.7%) for enhancing IGFBP-3, a major regulator of IGF-1 bioavailability, which has been inversely associated with IGF-1 signaling and is thought to exert context-dependent protective effects against tumorigenesis. The data suggest that silybin interventions could potentially attenuate PSA levels and influence growth factor pathways (IGF-1/IGFBP-3 axis) implicated in prostate cancer biology. However, heterogeneity in (i) study designs, (ii) extract formulations, (iii) dosing regimens, and (iv) follow-up durations limits definitive conclusions on clinical efficacy. It should also be stressed that biomarker endpoints, though relevant, are surrogate outcomes and may not directly translate to long-term clinical benefits such as progression-free survival.

**Figure 3:**
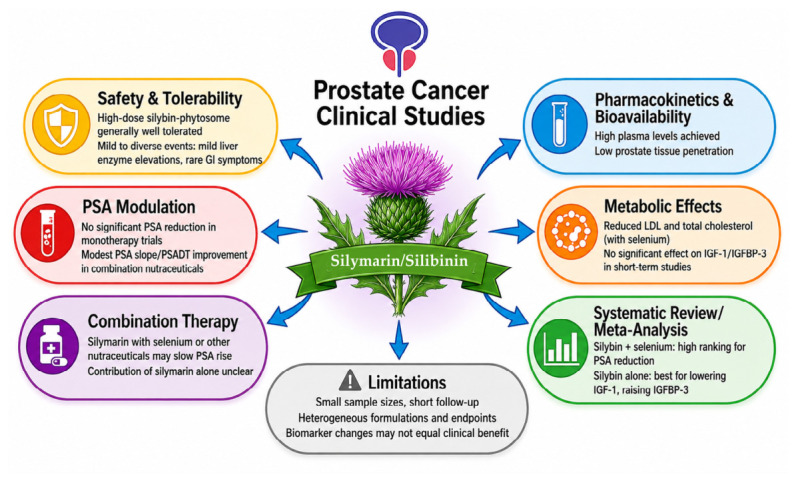
Summary of the effects and limitations observed in clinical studies with silymarin and silibinin.

Figure 3 provides an overview of the effects and limitations reported in clinical studies investigating silymarin and silibinin.

## 5. Discussion

The present review critically reassesses the current evidence regarding the biological and anticancer properties of *Silybum marianum* and its major bioactive constituents, with particular emphasis on their potential role in PCa. Although research interest in milk thistle-derived nutraceuticals has declined in recent years and robust clinical evidence remains limited, the available experimental literature still provides substantial evidence supporting their biological activity in PCa models. In particular, silymarin and silibinin have consistently demonstrated the ability to modulate multiple molecular pathways involved in prostate carcinogenesis, tumor progression, angiogenesis, metastatic dissemination, and treatment resistance. In light of the increasing interest in complementary and alternative medicine approaches in oncology, revisiting these compounds may therefore be relevant for identifying multitarget, low-toxicity adjunctive strategies deserving of further investigation. In this context, revisiting these compounds may be relevant within the broader and increasingly investigated field of complementary and integrative oncology, especially in the search for multitarget, low-toxicity adjunctive therapeutic strategies.

Extensive preclinical evidence indicates that silymarin and its main component, silibinin, are capable of modulating several hallmarks of cancer, including sustained proliferation, resistance to cell death, invasion, metastasis, and angiogenesis [76,78,82,84,96,97]. In multiple PCa models, such as LNCaP, DU145, PC3, and rat prostate cancer cell lines, these compounds inhibit proliferation and induce apoptosis through mechanisms involving cell cycle arrest, cyclins and CDKs modulation, and the activation of programmed cell death pathways [74,75,85]. Silibinin has also been reported to interfere with epithelial–mesenchymal transition, invasion, and angiogenesis [74,75,108]. Moreover, emerging findings suggest a possible role in epigenetic regulation and the reactivation of tumor-suppressive programs [133]. Collectively, these pleiotropic effects are consistent with the multifactorial pathogenesis of PCa and support the conceptual rationale for milk thistle-derived compounds in both prevention and disease modulation [74,108]. However, it should be emphasized that these mechanistic observations are derived predominantly from in vitro and animal studies, and their translational relevance in human PCa remains to be fully established.

Evidence from in vivo studies further supports these observations. In transgenic and xenograft models of prostate cancer, the dietary or pharmacological administration of silibinin significantly reduces tumor growth, angiogenesis, and metastatic dissemination without producing substantial systemic toxicity. These effects were associated with coordinated modulation of molecular regulators involved in proliferation, apoptosis, and vascularization, including VEGF, Bcl-2, survivin, and various cyclins and CDKs. Notably, silibinin also demonstrated chemopreventive activity in the TRAMP model, where long-term administration limited progression from prostatic intraepithelial neoplasia to invasive carcinoma [115,116,117,119]. It should also be noted that several preclinical studies employed silibinin doses that may exceed clinically achievable human exposure levels, partly owing to the compound’s unfavorable pharmacokinetic profile and limited tissue bioavailability.

Despite the promising preclinical evidence, translation into clinical practice remains limited, as the studies reviewed showed no robust clinical evidence supporting silibinin as an anticancer therapy for PCa. Current clinical studies do not provide robust evidence supporting silibinin or silymarin as effective anticancer therapies for PCa. Most available investigations are characterized by small sample sizes, heterogeneous study designs, and a predominant focus on safety, tolerability, pharmacokinetics, or surrogate biomarkers rather than clinically meaningful oncological endpoints such as progression-free survival or overall survival. Silybin-based formulations, particularly phytosomes, appear to be generally well tolerated, although mild and dose-dependent adverse effects have been reported [125]. Importantly, pharmacokinetic analyses performed in the study by Flaig et al. demonstrated that, although high systemic exposure to silibinin can be achieved, prostate tissue penetration remains poor, likely reducing its therapeutic potential [126].

Additional limitations arise from studies evaluating silymarin-containing nutraceutical combinations in patients with PCa. Although some reports described effects on PSA kinetics or metabolic parameters [128], interpretation is complicated by the concomitant administration of multiple bioactive compounds, including selenium, soy isoflavones, and lycopene [129,130,131], making it difficult to isolate the contribution of silymarin. Furthermore, variability in formulations, dosing regimens, and treatment duration contributes to the heterogeneity of currently available evidence. In addition, most studies rely on surrogate biomarkers rather than clinically meaningful endpoints such as disease progression or survival.

Systematic reviews and meta-analyses provide further, albeit heterogeneous, insights. While some data suggest that silibinin may influence pathways relevant to PCa progression, including PSA modulation and the IGF-1/IGFBP-3 axis, variability in study design, formulations, and treatment regimens limits the strength and generalizability of these findings [132].

Several challenges, therefore, remain to be addressed. The pharmacokinetic profile of silibinin, characterized by relatively low oral bioavailability and limited distribution to the prostate, represents a major limitation for clinical translation [134]. Advanced delivery systems, such as nanoparticles and phospholipid complexes, have been developed to improve absorption and bioavailability, although their impact on clinical outcomes remains uncertain [135]. Future studies should also focus on identifying specific molecular contexts in which these compounds may exert greater therapeutic benefit.

Importantly, the discrepancy between the extensive mechanistic evidence observed in preclinical settings and the paucity of well-designed clinical studies highlights a substantial translational gap in this field. Rather than necessarily reflecting a lack of biological potential, this gap may partly derive from the progressive shift in research priorities away from nutraceutical-based approaches despite their favorable safety profile and multitarget biological activity. In this regard, milk thistle-derived compounds remain of interest within the broader framework of complementary and integrative oncology, particularly as potential adjuncts to standard anticancer therapies.

Indeed, preclinical evidence suggests that silibinin and related compounds may enhance the activity of conventional anticancer agents and modulate mechanisms associated with therapeutic resistance, thereby supporting further investigation in multimodal treatment settings [101,102,105,106,107].

In summary, currently available evidence suggests that *Silybum marianum* derivatives possess biologically relevant anticancer properties in prostate cancer models. Nevertheless, given the limited evidence currently available regarding their effects on prostate cancer, adequately powered studies involving large patient cohorts, as well as investigations employing standardized and controlled formulations administered as single agents rather than in combination therapies, are warranted to more definitively establish their therapeutic potential and clinical efficacy.

## 6. Materials and Methods

A PubMed, Google Scholar, and Web of Science search was performed to collect the published data using the keywords milk thistle, *Silybum marianum*, phytochemicals, chemical composition, silymarin, silibinin, flavolignans, prostate cancer, in vitro, in vivo cancer prevention, and toxicology. Several websites and related articles were also incorporated. Additionally, some selected articles were manually searched. The inclusion criteria for this review encompassed systematic reviews and experimental studies on milk thistle or its main components, with no restrictions on the publication timeframe.

An in-depth literature search was also conducted to identify relevant human studies investigating the association between *Silybum*, *Silybum* phytochemicals, and PCa.

The literature search was performed across several electronic databases, including Medline, PubMed, and Embase, covering articles in English. A combination of Medical Subject Headings (MeSH) terms was used as a search strategy to ensure comprehensive retrieval of relevant literature. Key search terms included “Cancer*”, “Prostat*”, “Carcinoma*”, “Silybum”, “Thistle”, “Silymarin”, “Silybin”, “Silibinin”, etc.

Study identification, screening, eligibility assessment, and inclusion, as well as the drawing of PRISMA flowcharts (Figure S1), were performed according to the PRISMA guidelines [136].

## 7. Conclusions

Overall, the in vitro evidence reviewed here highlights a broad and multifaceted anticancer profile for silymarin and its major bioactive constituent, silibinin, in prostate cancer. These effects include cell cycle arrest, inhibition of proliferation, induction of differentiation, and suppression of invasive and metastatic behaviors. Mechanistically, silibinin modulates several key signaling pathways involved in prostate cancer progression, such as PI3K/Akt/mTOR, MAPK, JAK/STAT, and Wnt/β-catenin, and interferes with critical processes including epithelial–mesenchymal transition and tumor cell invasion. Despite these promising preclinical findings, clinical evidence remains limited and largely exploratory. Silymarin- and silibinin-based formulations consistently demonstrate a favorable safety profile. Still, their effects on PSA levels and tumor progression appear modest, context-dependent, and potentially influenced by formulation and dosing. However, more consistent biological effects have been observed in benign or post-surgical contexts, supporting a potential role as safe adjunctive agents for symptom management, modulation of inflammatory processes, stabilization of biomarkers, and possibly chemoprevention, rather than direct tumor control.

In conclusion, *Silybum marianum* and its bioactive flavolignans exhibit a range of biological activities that may interfere with key molecular mechanisms underlying prostate cancer development and progression. While preclinical data strongly support their anticancer potential, robust clinical validation is still lacking. Well-designed and adequately powered clinical trials are therefore needed to better define their therapeutic value, optimize formulations and dosing strategies, and clarify whether these compounds can meaningfully contribute to prostate cancer prevention or management.

## Figures and Tables

**Table 1:** Chemical structures of the major milk thistle constituents. Chemical structures were drawn with the PubChem Sketcher 2.4 web-based tool (https://pubchem.ncbi.nlm.nih.gov//edit3/index.html, accessed on 6 May 2026) based on the International Chemical Identifier (InChI) retrieved from PubChem (https://pubchem.ncbi.nlm.nih.gov/, accessed on 6 May 2026).

Silibinin (mixture of silybin A, shown here, and silybin B)	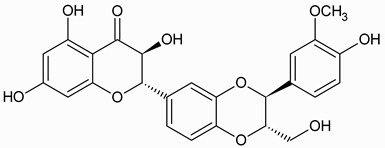
Isosilibinin (mixture of isosilybin A, shown here, and isosilybin B)	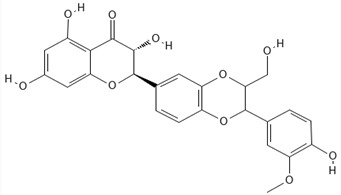
Silichristin (also known as silychristin)	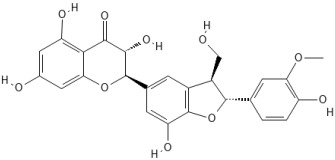
Isosilichristin (also known as isosilychristin)	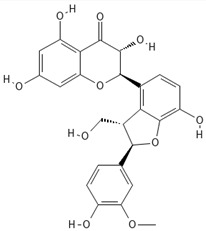
Silidianin (also known as silydianin)	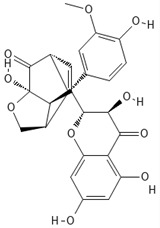

## Data Availability

No new data were created or analyzed in this study. Data sharing is not applicable to this article.

## Supplementary Materials

The following supporting information can be downloaded at https://www.mdpi.com/article/10.3390/ijms27104605/s1.
