# Supplementary material for: Silybum marianum-Derived Compounds in Prostate Cancer: Mechanisms of Action and Translational Potential
Source: Int J Mol Sci. 2026 May 20;27(10):4605. doi: 10.3390/ijms27104605 (PMC13206769; doi:10.3390/ijms27104605)
Supplement: Supplementary file 1 [file ijms-27-04605-s001.zip › ijms-4307553-supplementary.pdf]

Supplementary Materials

Silybum marianum-Derived Compounds in Prostate Cancer: Mechanisms of Action and Translational Potential

Federica Randisi [†,‡], Giulia Modoni [†,§], Mattia Riva [‖], Gianpaolo Perletti, Davide Odorico, Emanuela Marras and Marzia Bruna Gariboldi *

Department of Biotechnology and Life Sciences (DBSV), University of Insubria, 21100 Varese, Italy; frandisi1@uninsubria.it (F.R.); giulia.modoni@istitutotumori.mi.it (G.M.); mattia.riva@istitutotumori.mi.it (M.R.); gianpaolo.perletti@uninsubria.it (G.P.); dodorico@uninsubria.it (D.O.); emanuela.marras@uninsubria.it (E.M.)

* Correspondence: marzia.gariboldi@unisubria.it; Tel.: +39-0331339418

† These authors contributed equally to this work.

‡ Ph.D. student at the Life Science and Biotechnology Ph.D. course (University of Insubria, 21100 Varese, Italy).

§ Current address: Microenvironment and Biomarkers in Solid Tumors Unit, Fondazione IRCCS Istituto Nazionale dei Tumori di Milano, 20100 Milan, Italy.

‖ Current address: Molecular Pharmacology Unit, Department of Experimental Oncology, Fondazione IRCCS Istituto Nazionale dei Tumori di Milano, 20100 Milan, Italy.

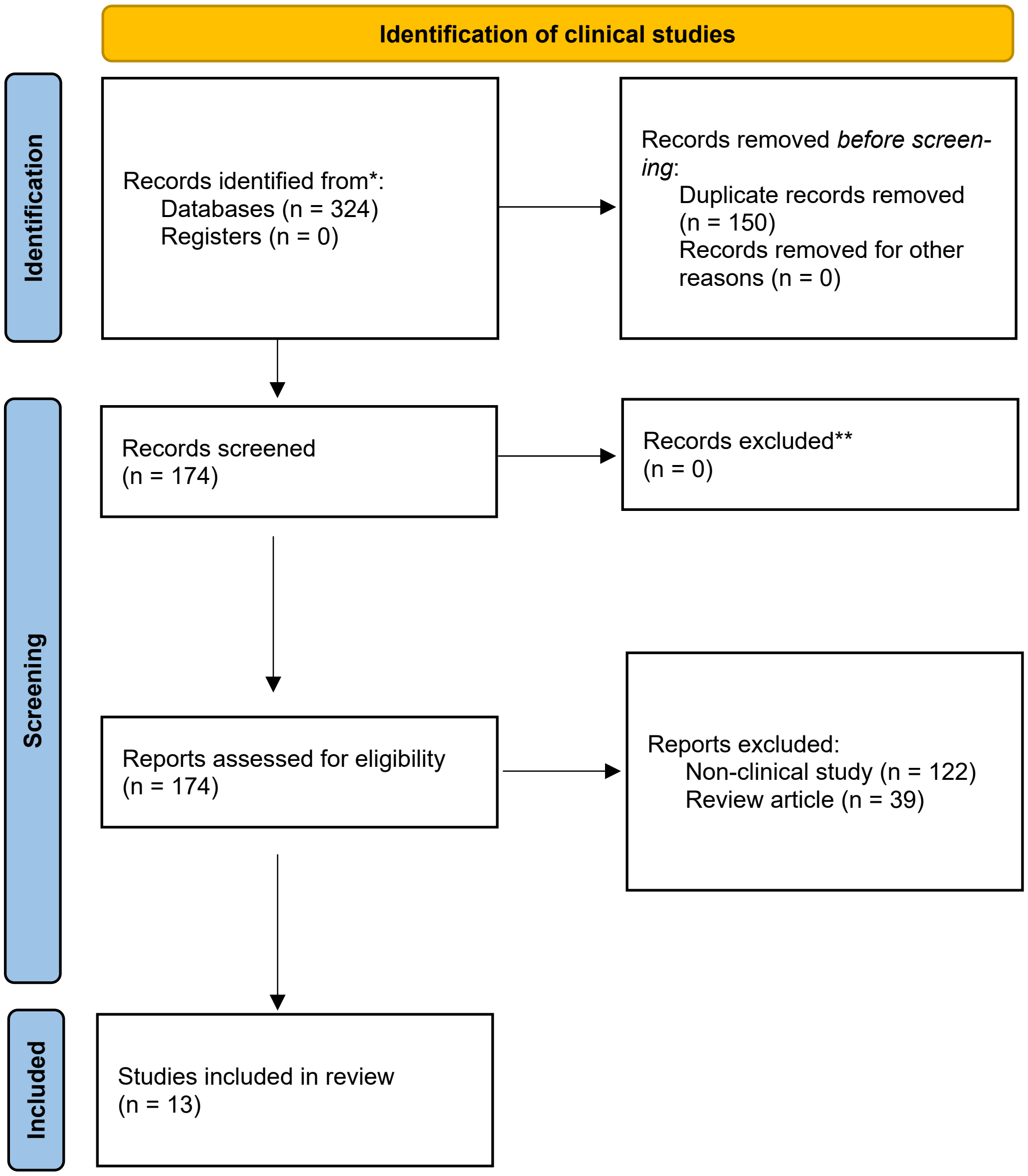

Figure S1. PRISMA flow-chart of the process of record retrieval and screening for human studies, with reasons for exclusion.
